# Identification of Wnt/β-Catenin- and Autophagy-Related lncRNA Signature for Predicting Immune Efficacy in Pancreatic Adenocarcinoma

**DOI:** 10.3390/biology12020319

**Published:** 2023-02-16

**Authors:** Hao Lyu, Jiahui Zhang, Qian Wei, Yuan Huang, Rui Zhang, Shuai Xiao, Dong Guo, Xing-Zhen Chen, Cefan Zhou, Jingfeng Tang

**Affiliations:** 1 Cooperative Innovation Center of Industrial Fermentation (Ministry of Education & Hubei Province), Hubei Key Laboratory of Industrial Microbiology, Hubei University of Technology, Wuhan 430068, China; 2 National “111” Center for Cellular Regulation and Molecular Pharmaceutics, Key Laboratory of Fermentation Engineering (Ministry of Education), Hubei University of Technology, Wuhan 430068, China; 3 Membrane Protein Disease Research Group, Department of Physiology, Faculty of Medicine and Dentistry, University of Alberta, Edmonton, AB T6G2H7, Canada

**Keywords:** long non-coding RNA, autophagy, Wnt/β-catenin, prognosis, pancreatic adenocarcinoma

## Abstract

**Simple Summary:**

Pancreatic adenocarcinoma (PAAD) is a devastating malignant tumor with a dismal prognosis. How to evaluate the prognosis efficiently is urgently needed for PAAD patients. Both activated autophagy and Wnt/β-catenin signaling pathway were reported to show significant relevancy with PAAD. This study aimed to identify the potential prognosis factor based on autophagy and Wnt/β-catenin related genes in PAAD. We identified 45 prognostic genes significantly associated with the overall survival of PAAD patients. Then a prognosis model was constructed according to six warlncRNAs (LINC01347, CASC8, C8orf31, LINC00612, UCA1, and GUSBP11). In conclusion, our results provide a basis for the pathogenesis of PAAD, including the cross-talk between autophagy and the Wnt/β-catenin signaling pathway, and reveal a prognostic indicator with the potential of the efficacy of prognosis and immunotherapeutic responses in PAAD patients.

**Abstract:**

Pancreatic cancer is one of the tumors with a poor prognosis. Therefore, it is significant and urgent to explore effective biomarkers for risk stratification and prognosis prediction to promote individualized treatment and prolong the survival of patients with PAAD. In this study, we identified Wnt/β-catenin- and autophagy-related long non-coding RNAs (lncRNAs) and demonstrated their role in predicting immune efficacy for PAAD patients. The univariate and multivariate Cox proportional hazards analyses were used to construct a prognostic risk model based on six autophagy- and Wnt/β-catenin-related lncRNAs (warlncRNAs): LINC01347, CASC8, C8orf31, LINC00612, UCA1, and GUSBP11. The high-risk patients were significantly associated with poor overall survival (OS). The receiver operating characteristic (ROC) curve analysis was used to assess the predictive accuracy of the prognostic risk model. The prediction efficiency was supported by the results of an independent validation cohort. Subsequently, a prognostic nomogram combining warlncRNAs with clinical indicators was constructed and showed a good predictive efficiency for survival risk stratification. Furthermore, functional enrichment analysis demonstrated that the signature according to warlncRNAs is closely linked to malignancy-associated immunoregulatory pathways. Correlation analysis uncovered that warlncRNAs’ signature was considerably associated with immunocyte infiltration, immune efficacy, tumor microenvironment score, and drug resistance.

## 1. Introduction

Pancreatic adenocarcinoma is a rapidly fatal disease and has a dismal prognosis. The incidence and mortality of PAAD rank fifth among digestive system tumors in China, of which the five-year survival rate is about 9% [1]. Existing therapy cannot considerably ameliorate the prognosis of PAAD patients, so surgery is still the basis for curing PAAD. The bulk of patients with PAAD (up to four-fifths) lose the chance for surgery, because the cancer is without effective early diagnosis, inability to resect, or highly malignant [2]. For patients with advanced PAAD, the effect of various therapies on survival is limited [3]. However, the molecular mechanisms underlying PAAD progression remain largely unknown. Therefore, it is of great significance and urgency to explore practical and innovative biomarkers to improve the early diagnosis efficiency and predict the prognosis of PAAD, thereby prolonging the survival of patients.

Autophagy is an evolutionarily conserved, programmed cellular process that eliminates damaged cellular organelles and misfolded proteins to maintain cellular homeostasis [4]. The process is controlled by a set of autophagy-related genes (ATGs) that regulate multistep autophagy. The deregulation of autophagy has been linked to numerous diseases, including cancer, neurodegeneration, cardiovascular disorders, and aging. Numerous studies have revealed that autophagy plays a crucial role in pancreatic tumor growth and progression.

It is well known that the intrinsic cause of pancreatic tumorigenesis is gene mutations, especially mutations in KRAS and TP53 [5]. It was reported that autophagy accelerated PDAC progression, driven by mutant KRAS [6]. Autophagy was also observed to inhibit PDAC onset, driven by the absence of oncogenic Kras and p53 in mice [7]. Moreover, the studies of patient-derived xenografts (PDX) formed in immuno-compromised mice showed that inhibition of autophagy impaired tumorigenesis irrespective of p53 status [8]. According to existing research, the crucial role of autophagy in pancreatic cancer is beyond doubt. Consequently, studying new autophagy-related biomarkers to improve early diagnosis and prognosis is promising for PAAD patients.

The Wnt/β-catenin pathway controls myriad biological phenomena ranging from cancer and development to early animal evolution [9]. In the Wnt/β-catenin or canonical Wnt signaling pathway, the current research mainly focused on, β-catenin is phosphorylated by CK1 and APC/Axin/GSK-3β complex when the Wnt ligand is absent and then used as a target to cause ubiquitination and proteasome degradation through the β-TrCP. While in the presence of Wnt ligands, Wnt ligands bind to Frizzled (FZD) receptors and then form a larger complex with LRP5/6, recruiting Dishevelled (Dvl) protein and blocking the formation of the Axin/GSK3/APC complex, β-catenin escapes degradation in the APC/Axin/GSK-3β complex [9,10]. The Wnt/β-catenin pathway was reported to be activated in the pancreatic cancer mouse model, and the inhibition of the Wnt/β-catenin pathway blocked the proliferation and induced apoptosis of cultured PDAC cells [11]. The Wnt/β-catenin signaling pathway promotes the proliferation of pancreatic cancer cells and plays an essential role in gemcitabine resistance to pancreatic cancer [4,12]. The accumulating evidence supports Wnt/β-catenin-mediated pancreatic cancer cell survival and the therapeutic potential for the Wnt/β-catenin pathway. Therefore, the Wnt/β-catenin pathway can be used as an effective prognostic prediction strategy for pancreatic cancer.

Long non-coding RNAs (lncRNAs) are transcripts of over 200 nucleotides with no or limited potential to code proteins. The main function of lncRNAs not only affects epigenetic modification, transcriptional activation, translation, and post-translational modification but also plays a crucial role in tumorigenesis, metastasis, prognosis, and diagnosis [13,14]. Numerous studies have demonstrated that dysregulated expression of lncRNAs is correlated with various cancer stages and drug resistance, including PDAC [15,16,17]. Previous studies also showed that lncRNA markedly participated in regulating autophagy and Wnt/β-catenin [5,18,19,20]. The cross-talk mechanism between autophagy and Wnt/β-catenin synergistically promotes the progression of pancreatic cancer and deserves further exploration. Therefore, it is appropriate and accurate to construct a prognostic model by combining autophagy and the Wnt/β-catenin pathway.

Herein, we first analyzed differentially expressed lncRNAs associated with autophagy factors and Wnt/β-catenin-related genes. Then, the risk model with six lncRNAs based on the TCGA data and corresponding clinical information was constructed. The PAAD patients were divided into high- and low-risk groups according to their risk scores. Subsequently, the various factors of the high- and low-risk groups were compared, including immune efficacy, immune infiltration, drug sensitivity, and biological function.

## 2. Materials and Methods

### 2.1. Datasets and Sample Extraction

RNA-seq data and corresponding clinical information of the PAAD in the training and validation cohorts were downloaded from the Cancer Genome Atlas (TCGA, https://portal.gdc.cancer.gov/) and International Cancer Genome Consortium (ICGC, https://dcc.icgc.org/) databases, respectively. The RNA-seq data of 167 adjacent normal tissue samples were obtained from the Genotype-Tissue Expression (GTEx) database. A total of 167 PAAD patients with complete clinicopathological data were included in subsequent analyses. R software version (https://www.r-project.org/): 4.1.1 was used for all analyses in the study.

### 2.2. Identification and Screening of Differentially Expressed arlncRNAs and wrlncRNAs

The autophagy gene was obtained from The Human Autophagy Database (HADb, http://www.autophagy.lu/index.html (accessed on 4 May 2022)). One hundred and seventy Wnt/β-catenin signaling pathway genes were collected from the Kyoto Encyclopedia of Genes and Genomes (KEGG) database https://www.genome.jp/kegg/ (accessed on 5 June 2022)). The “DEseq2” R package was used for screening the DEGs between 178 PC samples from TCGA and 171 normal samples (4 from TCGA and 167 from GTEx) [21], with a false discovery rate (FDR) < 0.05 and |log2 fold change (FC)| >1.5. The Pearson correlation analysis was performed by R to screen the arlncRNAs or wrlncRNAs. In this study, the correlation coefficient |R| > 0.4 and *p* < 0.001 were considered statistically significant.

### 2.3. Construction and Validation of the warlncRNAs Prognostic Signature

In total, 354 warlncRNAs were filtrated using univariate COX regression analysis, and 45 warlncRNAs were significantly associated (*p* < 0.05) with OS by Kaplan–Meier survival analyses. Then, the selected warlncRNAs were subjected to LASSO regression analysis. The warlncRNAs obtained from LASSO regression were used to screen the optimal warlncRNAs to construct the prognostic signature by applying multivariate Cox regression analysis. A prognostic signature was developed based on the coefficient values and expression levels of six warlncRNAs (LINC01347, CASC8, C8orf31, LINC00612, UCA1, and GUSBP11), with risk scores = ΣCoef × exp (genes).

### 2.4. Immune Infiltration Analysis and Efficacy Prediction

The estimation of stromal and immune cells in malignant tumor tissues using expression data (ESTIMATE) was used to calculate the immune, stromal, and ESTIMATE scores between high- and low-risk PAAD patients by the “estimate” package. Finally, cell type identification by estimating relative subsets of RNA transcripts (CIBERSORT) was used to estimate the abundance of member cell types based on gene expression data in a mixed cell population. The expression of 47 immune checkpoints in the high- and low-risk groups was analyzed to evaluate the differences in immune efficacy [22].

### 2.5. Statistical Analysis

All computational and statistical analyses were conducted using R. The LASSO, survival, and ROC analyses were performed using the “glmnet”, “survival”, and “survivalROC” package [23], respectively. The function analysis was performed by the “clusterProfiler” package [24]. The Kruskal–Wallis test was used to compare differences between groups. Statistical significance was set at *p* < 0.05 and a false discovery rate (FDR) of q < 0.05 (except where specially noted).

## 3. Results

### 3.1. Identification of Differentially Expressed Autophagy- and Wnt/β-Catenin- Related lncRNAs in PAAD

**Figure 1:**
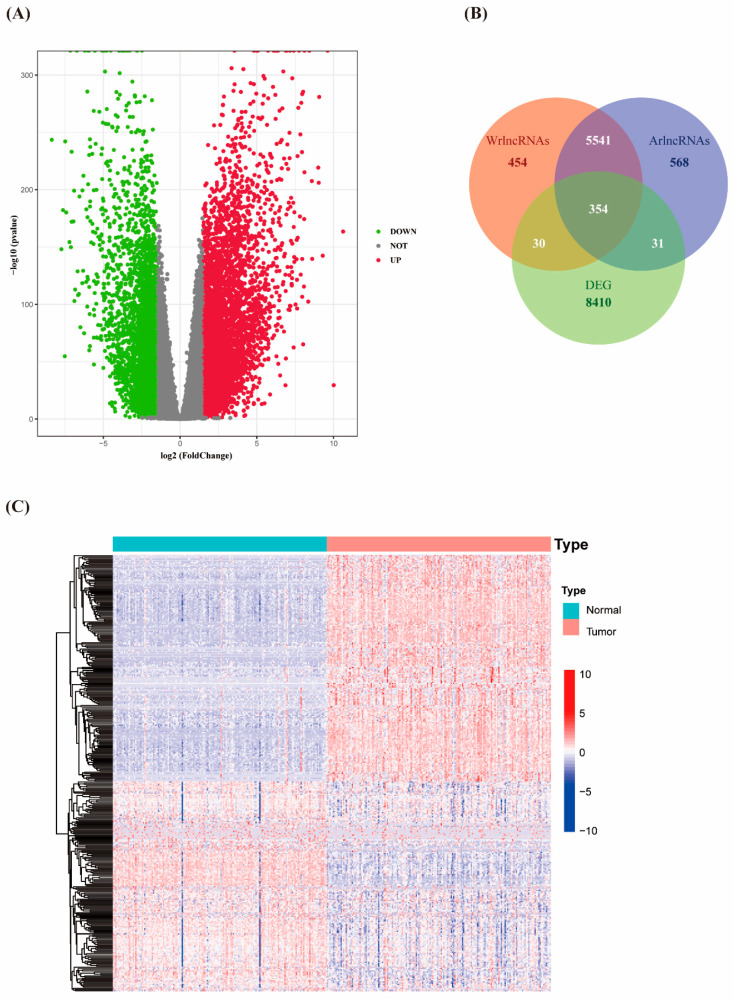
Identification of differentially expressed warlncRNAs in PAAD. (**A**) Volcano plot of DEGs between PAAD and adjacent normal tissue. (**B**)Venn diagram of wrlncRNAs, arlncRNAs, and DEGs intersection. (**C**) Heatmap of the expression levels of 354 candidate warlncRNAs.

Differential gene analysis was performed to identify differentially expressed genes (DEGs) between 178 PC tissues and 171 normal tissues using RNA-seq expression data obtained from TCGA and GTEx. A total of 8825 DEGs were gained, including 4738 upregulated and 4087 down-regulated DEGs (Figure 1A). The expression data of 14,072 lncRNAs were extracted from PAAD tumor tissues in the TCGA database. It was defined as the coexpression relationship if the Pearson correlation analysis result between lncRNA and autophagy genes is significant. Conclusively, 6494 autophagy-associated lncRNAs (arlncRNAs) were screened. Moreover, 6379 Wnt/β-catenin related lncRNAs (wrlncRNAs) were obtained using the same method and criteria. Subsequently, the intersection of DEGs, arlncRNAs, and wrlncRNAs was evaluated, and 354 autophagy- and Wnt/β-catenin-related lncRNAs (warlncRNAs) were obtained (Figure 1B,C, Supplementary Table S1).

### 3.2. Construction of the Prognostic Risk Score Model of warlncRNAs

**Figure 2:**
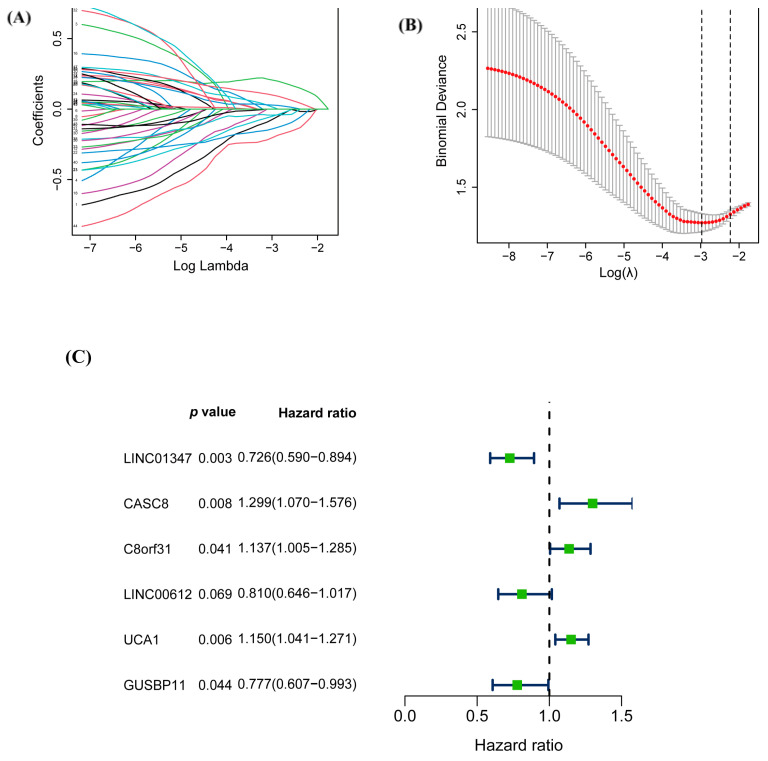
Construction of a prognostic signature based on warlncRNAs in PAAD patients. (**A**,**B**) The LASSO regression analysis (**A**) and cross-validation (**B**) were used to screen warlncRNAs. (**C**) The multivariate Cox regression analysis result indicated six warlncRNAs were identified to construct a warlncRNAs signature.

Univariate Cox analysis and Kaplan–Meier survival analysis were performed to screen 45 prognostic warlncRNAs with *p*-value < 0.05 (Supplementary Table S2). To further filter out the candidate lncRNAs, nine more rigorous warlncRNAs were identified according to 1000 repetitions of LASSO regression (Figure 2A,B). The remaining warlncRNAs showed that 6 of the 9 were the independent prognostic signatures of PAAD using multivariate Cox analysis (Figure 2C). The prognosis risk score for each patient was calculated based on the expression of each warlncRNAs multiplied by the coefficient.

### 3.3. Survival Analysis and Expression Level in PAAD of warlncRNAs

**Figure 3:**
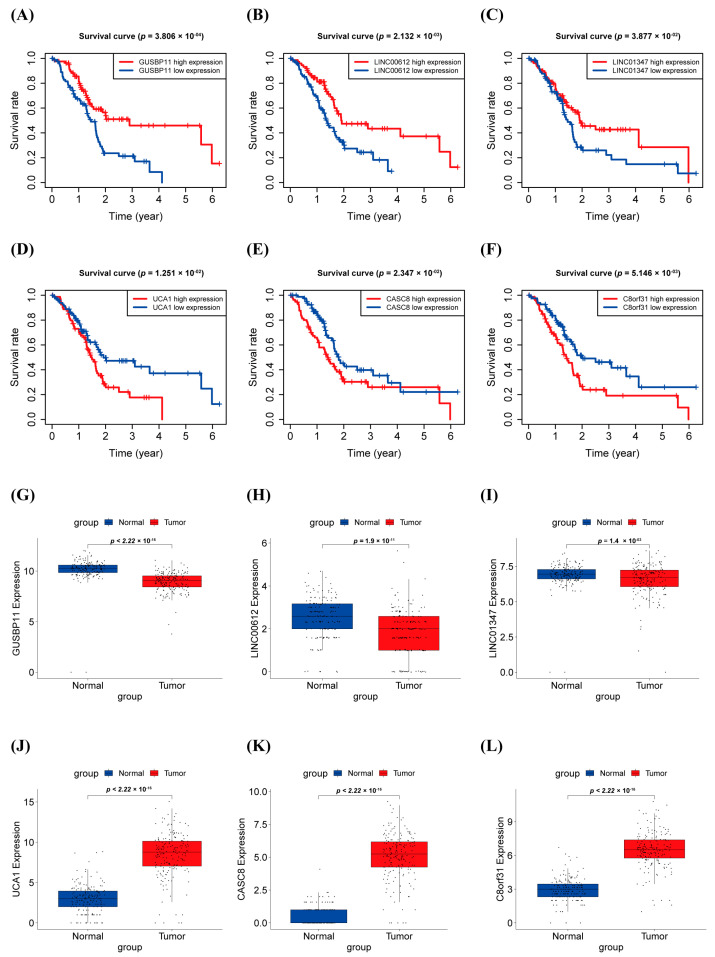
Survival curve and expression level in PAAD of warlncRNAs. (**A**–**F**) Kaplan–Meier survival curves for six prognostic warlncRNAs (all *p* < 0.05). (**G**–**L**) The expression level of six prognostic warlncRNAs in Normal and PAAD patients.

The OS analysis result of warlncRNAs indicated that the high expression of GUSBP11, LINC00612, and LINC01347 was positively correlated with longer overall survival of patients with PAAD (Figure 3A–C), while the high expression of CASC8, C8orf31, and UCA1 was positively associated with shorter overall survival of patients with PAAD (Figure 3D,E). The expression level of warlncRNAs was consistent with the result of the OS analysis. The expression levels of GUSBP11, LINC00612, and LINC01347 were significantly lower in PAAD than in normal tissue (Figure 3G–I), but CASC8, C8orf31, and UCA1 were inverse (Figure 3J–L). These results implied that GUSBP11, LINC00612, and LINC01347 were the protective factors in PAAD, while CASC8, C8orf31, and UCA1 were the carcinogenic factors in PAAD.

### 3.4. Prognostic Value of warlncRNAs Signature

**Figure 4:**
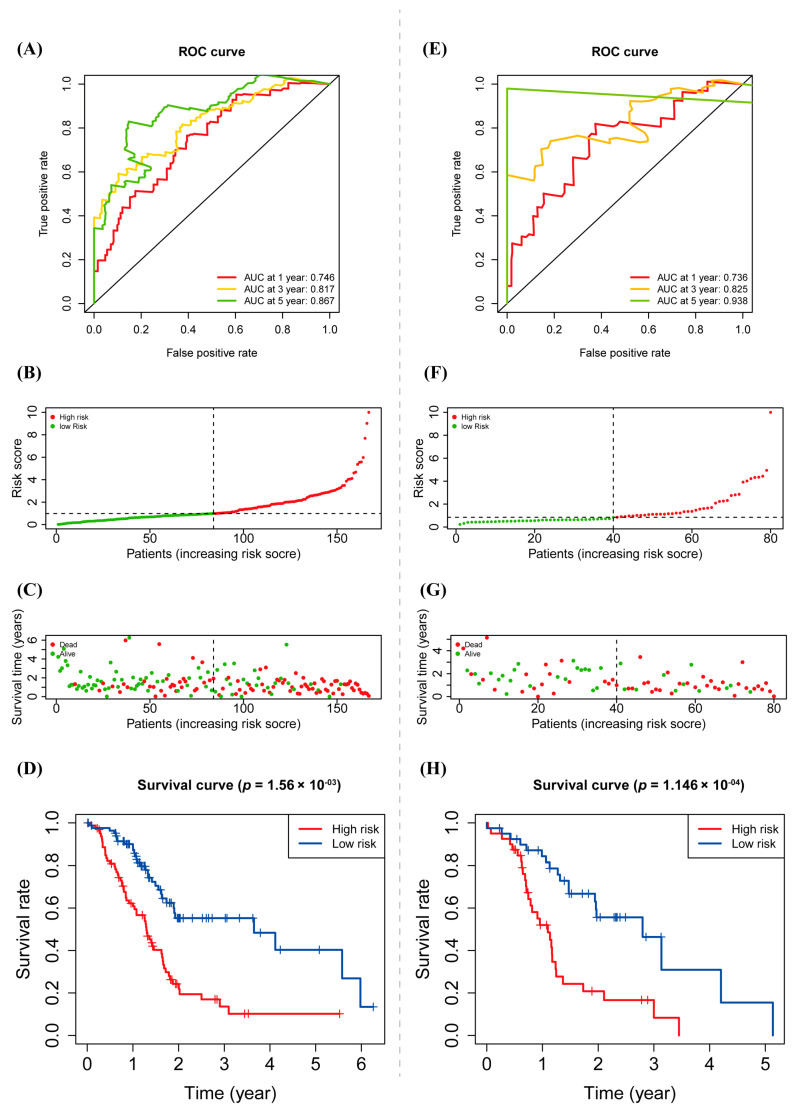
Prognostic value of warlncRNAs signature. (**A**) Time-dependent ROC curves of OS at 1-, 3-, and 5-year with other common clinical features in the training cohort. (**B**,**C**) Risk scores (**B**) and survival time (**C**) of each sample in the training cohort. (**D**) Kaplan–Meier analysis for OS of PAAD patients based on the risk group in the training cohort. (**E**–**H**) Prognostic value of warlncRNAs signature in the validation cohort.

The prediction ability of the risk model based on warlncRNAs signature is superior as the area under the curve (AUC) values of 3 years shown, which all were over 0.746 (Figure 4A). The scatter diagrams were visualized to show the risk scores, risk groups, survival status, and survival time of each PAAD patient (Figure 4B,C). Then the PAAD patients were divided into high-risk and low-risk groups according to the medium value of the risk score. Kaplan–Meier survival analysis showed a statistical significance in OS between two subgroups: the OS of the patients with low-risk scores was higher than that of those with high-risk scores (Figure 4D). Similar results were obtained in the validation cohort (Figure 4E–H).

**Figure 5:**
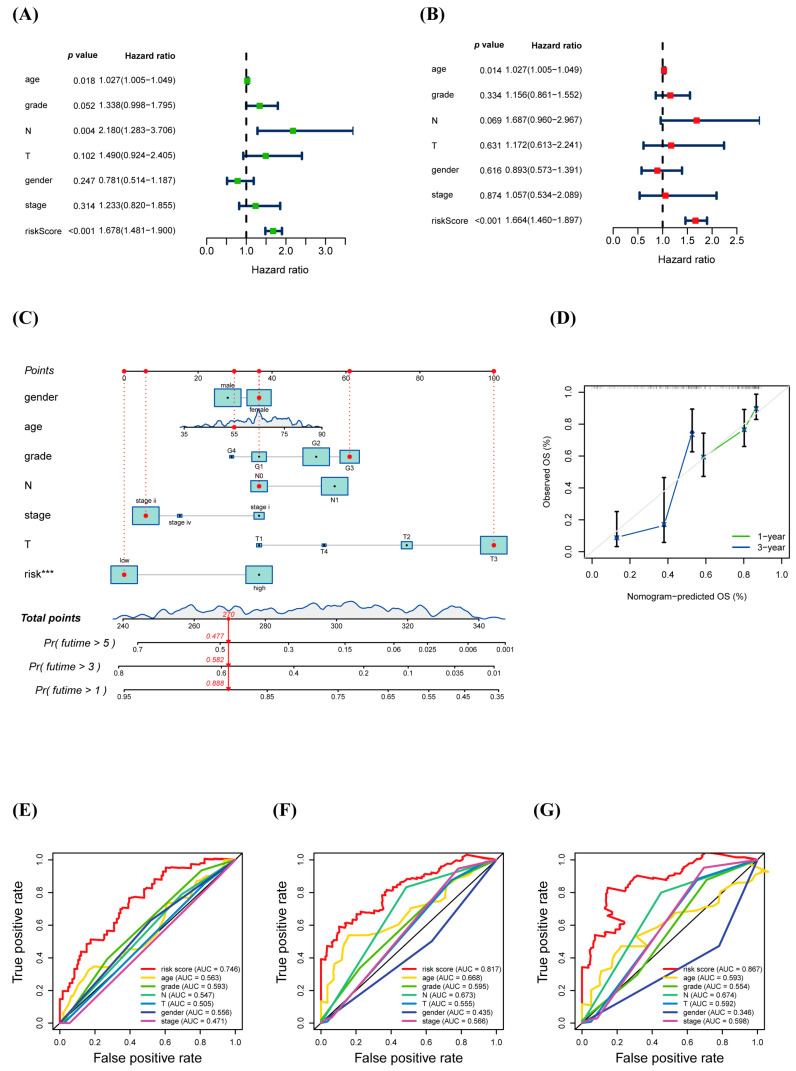
Effects of the risk score model and clinical parameters on the prognosis of PAAD patients. (**A**,**B**). Identification of the parameters related to OS by univariate (**A**) and multivariate Cox analyses (**B**) in the TCGA training cohort. (**C**) A clinical prognostic nomogram for predicting the 1-, 3-, and 5-year OS of patients with PAAD in TCGA training cohort. (**D**–**F**) A comparison of 1- (**D**), 3- (**E**), and 5-year (**G**) ROC curves with other common clinical traits in the TCGA training cohort. *** *p* ≤ 0.001.

Correlations between prognostic values and traditional clinical features were also examined. The results showed that age was also a slightly significant prognostic factor. Still, the risk score had the most substantial prognostic value in both the univariate and multivariate Cox regression analyses (Figure 5A,B). A clinically adaptable nomogram plot was drawn to estimate the 1-, 3-, and 5-year survival probabilities of patients with PAAD using warlncRNAs combined with other clinical traits (Figure 5C). The total clinical score depended on summing each item’s scores, which contributed to the survival prediction. The calibration curves of the nomogram for 1-year and 3-year indicated that the mortality predicted by the nomogram was close to the observed mortality (Figure 5D). Time-dependent ROC curves of 1-, 3-, and 5-year OS were performed, and the results showed that the AUC value for the risk score was significantly higher than other clinical factors for age, gender, tumor stage, T stage, and N stage (Figure 5E–G), further supporting the discerning capability of warlncRNAs in conjunction with clinicopathological factors for predicting survival in PAAD.

### 3.5. Construction of lncRNA–mRNA Network and Function Enrichment Analysis

**Figure 6:**
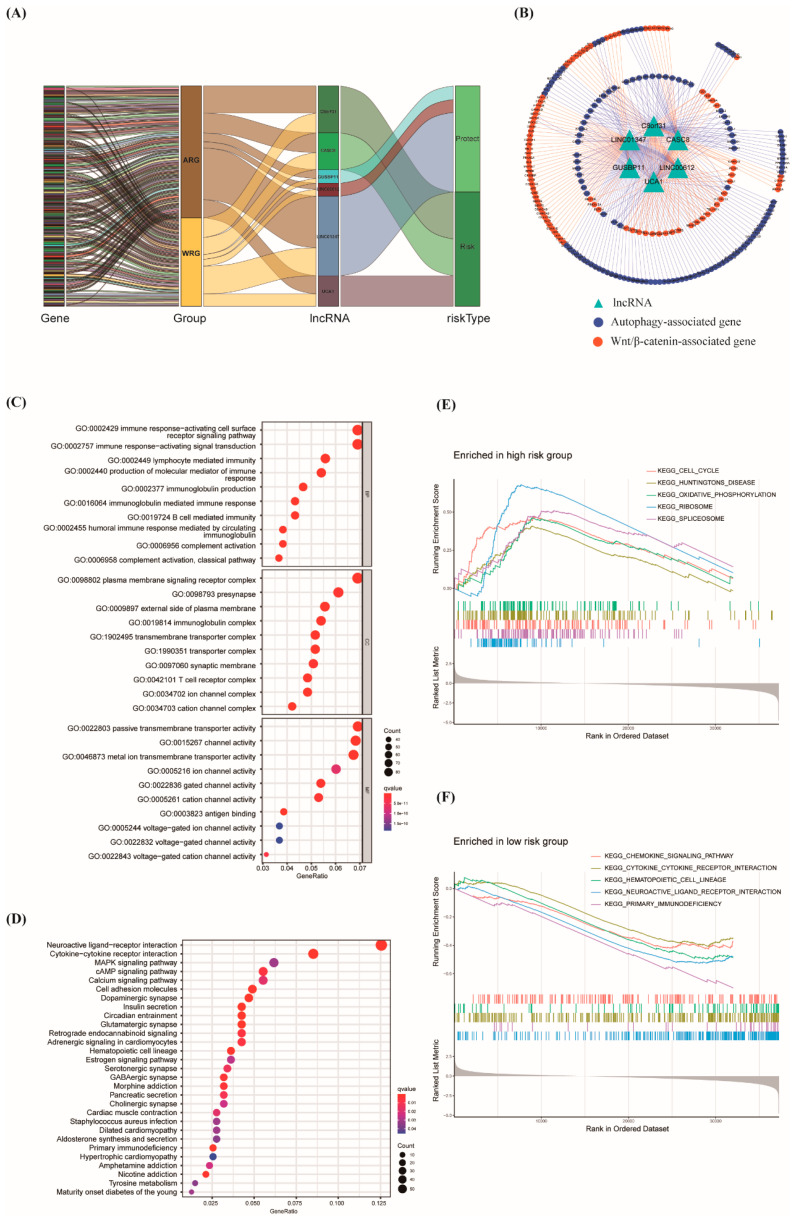
Construction of lncRNA-mRNA coexpression network and GO and KEGG enrichment analysis. (**A**) A Sankey diagram showed the co-occurrences of lncRNAs, mRNAs, and characters based on the risk score. (**B**) The lncRNA-mRNA network between six warlncRNAs and relevant mRNAs. (**C**) GO analysis of biological processes, cell components, and molecular functions based on the differentially expressed genes between the high- and low-risk groups. (**D**) The significantly enriched pathways of KEGG analysis based on the differentially expressed genes between the high- and low-risk groups. (**E**,**F**) The active biological pathways of GSEA shown in high- (**E**) and low-risk (**F**) groups. ARG: autophagy-related gene. WRG: Wnt/β-catenin related gene.

A total of 211 autophagy-related genes and 157 Wnt/β-catenin-related genes were found to be associated with six prognosis-related lncRNAs. Meanwhile, we established a lncRNA-mRNA coexpression network based on the relationship between these genes and lncRNAs and used Cytoscape (version 3.9.1, https://cytoscape.org/) and Sankey diagrams to visualize the network (Figure 6A,B).

The results of the top 10 enrichment terms in each category by gene ontology (GO) enrichment analysis between high- and low-risk groups are shown in Figure 6C. The terms related to immune function were significantly enriched in the biological process category, such as immune response-activating cell surface receptor signaling pathway, immune response-activating signal transduction, and lymphocyte-mediated immunity. While the bubble map of KEGG enrichment analysis also showed that the top 5 pathways are closely related to tumor progression, including cytokine-cytokine receptor interaction, MAPK signaling pathway, and cell adhesion molecules (Figure 6D). Gene set enrichment analysis (GSEA) was performed to identify the differentially active signaling pathway between the high- and low-risk groups. As shown in Figure 6E, potential pathways associated with tumorigenesis were activated in the high-risk groups. Conversely, multiple defensive pathways, such as immune-related signaling pathways, were enriched in the low-risk group (Figure 6F). These data also confirmed the validity of the prognostic model.

### 3.6. Immune Infiltration Analysis and Efficacy Prediction of warlncRNAs Signature

**Figure 7:**
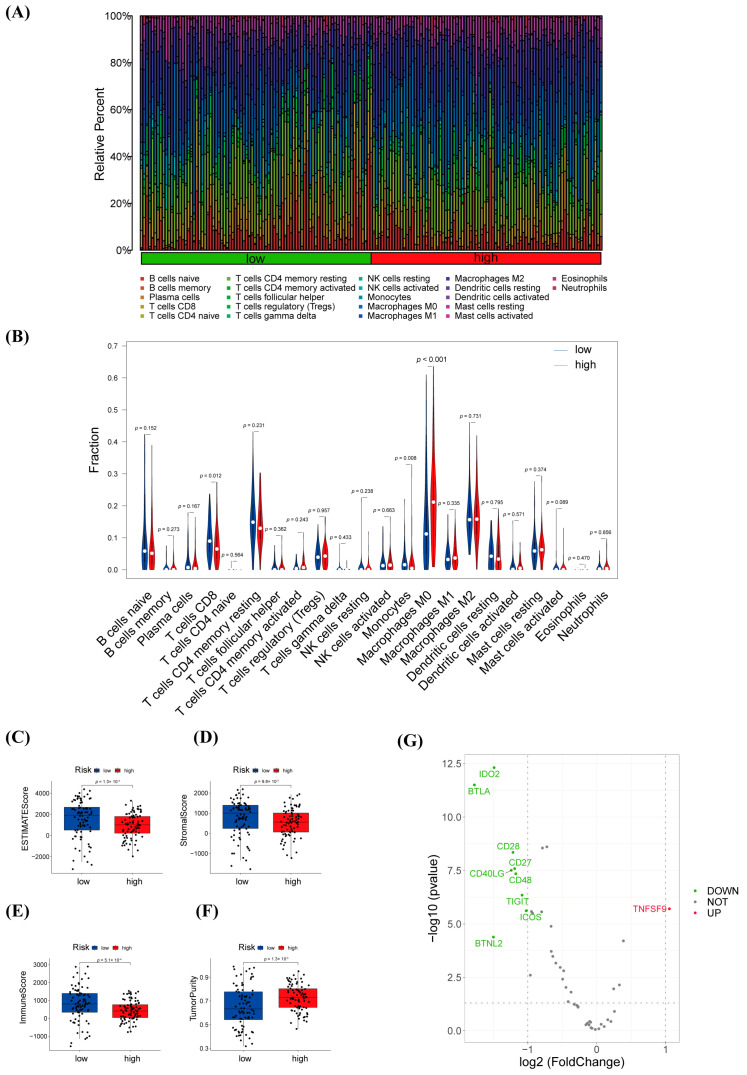
Identification of immunotherapeutic response based on warlncRNAs signatures. (**A**,**B**) The percentage proportion (**A**) and infiltration (**B**) of 22 immune cells between high- and low-risk groups in the training cohort. (**C**–**F**) Differential analysis of ESTIMATE (**C**), stromal (**D**), immuneScore scores (**E**), and tumor purity (**F**) according to ESTIMATE analysis between high- and low-risk populations in the training cohort. (**G**) The differential expression of 47 immune checkpoints between the high- and low-risk groups.

We calculated the percentage in the high- and low-risk subgroups of various immune cells by performing an immune cell infiltration analysis (Figure 7A). T cells CD8, monocytes, and macrophages M0 had apparent correlations with the risk score (Figure 7B). According to the ESTIMATE analysis, the scores for ESTIMATE, immune, and stromal were significantly higher in the low-risk group than in the high-risk population (Figure 7C–E). However, the tumor purity in the low-risk group is inverse (Figure 7F).

The reason for the difference in immune efficacy was preliminarily analyzed based on the differential expression of immune checkpoints. As the result indicated, nine genes were downregulated in the high-risk group, while TNFSF9 was upregulated (Figure 7G).

### 3.7. Differences in Response to Chemotherapy between High-Risk and Low-Risk Groups

**Figure 8:**
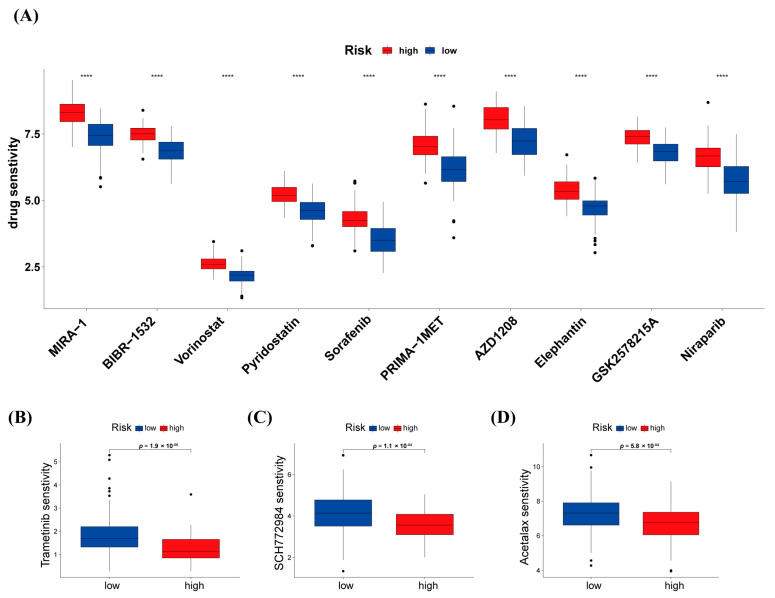
Prediction of drug sensitivity based on warlncRNAs signatures. (**A**) The top ten drugs with resistance in high-risk populations in the training cohort. (**B–D**) The potential drugs could contribute to the therapy of PAAD patients in the low-risk group. **** *p* ≤ 0.0001.

The prediction of the response to chemotherapy drugs in high- and low-risk groups was investigated by “oncoPredict”. The result showed that 159 commonly utilized chemotherapy and molecular targeted drugs had significant sensitivities difference, among 156 were found to have poorer efficacy in the high-risk group compared to the low-risk group. The targets of the top ten drugs with poorer efficacy were most associated with p53, KRAS, and autophagy (Figure 8A). The resistance to chemotherapy and targeted therapy of patients in the high-risk group can also be one of the reasons explaining the poor prognosis of patients with high-risk scores. Furthermore, the other three chemotherapy drugs were found to be potential compounds that may be used for the therapy of low-risk group PAAD patients: trametinib, SCH772984, and acetalax (Figure 8B–D).

## 4. Discussion

PAAD is a highly malignant digestive tumor associated with high morbidity and a poor prognosis [2]. Early diagnosis, accurate prediction of tumor progression, and efficacious intervention were the instant major challenges of PAAD. The carbohydrate antigen (CA19-9) is a typical diagnostic biomarker but is not applied to specifically and sensitively diagnose PAAD patients [25]. Moreover, compared with single clinical biomarkers, incorporating preclinical findings and molecular-guided therapies into a single model can significantly improve the accuracy of prognosis prediction and guide individualized therapy. Thus, it is significant and urgent to identify efficient and innovative biomarkers.

Autophagy is a conservative metabolism pathway that maintains cellular homeostasis by eliminating damaged organelles and macromolecules. Autophagy plays a dual role in cancer development due to the type, stage, or genetic context of cancers [26]. In the early stage of tumorigenesis, autophagy prevents tumor initiation and suppresses cancer progression as a survival pathway and quality-control mechanism [27,28]. It had been shown that beclin 1, a key protein for autophagy initiation, inhibited MCF7 cellular proliferation, in vitro clonogenicity, and tumorigenesis in nude mice by promoting autophagy activity [29]. Another study also identified that beclin 1^−/−^ caused embryonic death in mice, while beclin 1^+/−^ mutant mice suffer from a high incidence of spontaneous tumors [30]. The study in mice had shown that liver-specific knockout of ATG7 develops into hepatocellular adenocarcinoma [31]. The specific knockout in the pancreas of Atg5 or Atg7, the essential factor for autophagy, also causes the emergence of KRAS^G12D^-driven pre-malignant pancreatic lesions [7,8]. While in the late stage of tumorigenesis subjected to environmental stresses, autophagy, as a recycling system of bioenergetic components, contributes to the survival and growth of the established tumors and promotes the aggressiveness of the cancers by facilitating metastasis. It was shown that the knockdown of p62/sequestosome 1 (SQSTM1) strongly inhibited the growth of TSC2-null xenograft tumors [32]. The ablation of FIP200, a key factor for autophagy initiation, suppresses mammary tumor initiation and progression in a mouse model of PyMT-driven breast cancer by inhibiting autophagy [33].

The role of Wnt signaling in colorectal cancer has most conspicuously been described, but many other cancers also observed the aberrance of Wnt signaling. Although elevated expression of GSK-3β was observed, abnormal nuclear localization of β-catenin was also monitored in pancreatic adenocarcinoma [34]. FAM83A-dependent Wnt/β-catenin activation can enhance the expression of TSPAN1 to activate autophagy and promote the proliferation of pancreatic cancer cells [4].

Currently, the essential roles of lncRNAs in PAAD have been found, and are indispensable for tumorigenesis function, especially autophagy and Wnt/β-catenin signaling. The research data revealed that Lnc-PFAR induced autophagy by downregulating miR-141 and facilitated pancreatic stellate cells (PSCs) activation and pancreatic fibrosis [35]. lncRNA PVT1 was found to enhance gemcitabine resistance of pancreatic cancer not only by accelerating assembly of the autophagy-specific complex I (PtdIns3K-C1) to activate autophagy but also by activating the pygo-mediated Wnt/β-catenin pathway [12]. The PVT1-miR-20a-5p-ULK1 axis also promoted cytoprotective autophagy and cell growth [36].

Existing research has concentrated on the prognostic prediction of autophagy-associated lncRNAs in PAAD, and a number of lncRNA signatures linked to PAAD have been described. The cross-talk of autophagy and the Wnt/β-catenin pathway in PAAD has been supported by a growing body of research. It is necessary to identify the autophagy- and Wnt/β-catenin-related lncRNA to construct the prognostic prediction model for making great efficiency on the tumorigenesis, progression, and prognosis of cancer. In the study, a prognostic model based on six warlncRNAs was constructed (LINC01347, CASC8, C8orf31, LINC00612, UCA1, and GUSBP11) (Figure 2A). LINC01347 contributes to the 5-FU chemotherapy resistance of colorectal cancer and suppresses trophoblast cell migration, invasion, and EMT [37,38]. LINC00612 enhances the proliferation and invasion ability in cancer [39,40]. GUSBP11 plays a different role in tumor progression [41,42]. C8orf31 has identified a pathogenicity marker in breast cancer [43]. Multiple functions of UCA1 in cancer have been widely reported [44,45,46,47,48]. CASC8 is an important factor in promoting the proliferation and chemoresistance of cancer cells [49,50,51]. However, the role of six warlncRNAs other than UCA1 in autophagy or the Wnt/β-catenin pathway has not been reported. All patients were distinguished into high- and low-risk groups based on the median risk score. Unsurprisingly, the OS of patients with higher risk scores was poorer than low-risk scores both in the training cohort and the validation cohort (Figure 4D,H). Then the time-dependent AUCs of the 1-, 3-, and 5-year OS rates were 0.746, 0.817, and 0.867 (Figure 4A), which indicated that the predictive ability of the risk score model is more considerable than the previous report, which only used autophagy-associated lncRNAs [52]. Moreover, the risk score was superior to age in terms of being considered an independent prognostic factor by uni- and multivariate Cox regression analysis (Figure 5A,B). The nomogram is expected to improve clinical practice and guide the development of treatment strategies (Figure 5C). The GO and, subsequently, the functional enrichment analysis, including GO, KEGG, and GSEA, were performed, and the results showed that pathways that were mostly enriched were significantly associated with tumor progression and immunity (Figure 6C–F).

The increasing studies have demonstrated that the pivotal role contributed to the tumor progression of the tumor microenvironment. Consequently, it is obvious effects on the immunotherapeutic efficacy of patients to assess the tumor infiltrates. In this study, we used “CIBERSORT” to assess immune cell infiltration between high- and low-risk groups. Patients with high-risk scores had significant differences in the proportions of T cells CD8, monocytes, and macrophages M0, confirming the roles of warlncRNAs in the regulation of tumor immune infiltration (Figure 7B). In the study, the result of the immune checkpoints analysis showed that patients in the high-risk group had downregulation of multiple immune checkpoints, which may provide insight into tumor immunotherapy (Figure 7G).

In this study, the distinction of patients based on risk scores led to personalized treatment strategies. Furthermore, the risk stratification of patients has been confirmed to predict the response to chemotherapy. The results of drug sensitivity analysis indicated that patients with PAAD in the high-risk group benefited less from multiple chemotherapy agents (Figure 8A). The targets of the top ten drugs were mostly linked to p53, KRAS, and autophagy, which played key roles in tumorigenesis in PAAD and also suggested the predicted efficacy of warlncRNAs signature. We found that the high-risk group patients were more sensitive to trametinib, SCH772984, and acetalax (Figure 8B). Briefly, the result can provide a more reasonable therapeutic procedure to improve the survival rate of patients with PAAD.

By and large, compared to the classical clinical features including age, gender, and tumor stage (T stage and N stage), the risk score of warlncRNAs signature could be a robust prognostic factor to forecast clinical outcomes for PAAD patients. The results of univariate, multivariate Cox regression showed that decisions based on the risk score had more predominant effects than classical clinical factors. Furthermore, it is obvious that the warlncRNAs signature is more conducive to personalized treatment for high- and low-risk PAAD patients according to ROC, calibration curves, nomograms, functional enrichment analysis, and drug susceptibility, combined with the infiltration of immune cells. Admittedly, our study had several limitations. First, we constructed the training cohort from the TCGA database and used PAAD samples from the ICGC database as an external validation cohort. Nevertheless, the credibility of the results is required to identify via further experimental studies. Second, more prognostic factors, such as chemotherapy data and stage M, were not included since the clinical data in the existing PAAD database were incomplete. In addition, the molecular mechanisms underlying the effects of warlncRNAs should be elucidated by experiments in vivo and in vitro.

## 5. Conclusions

In conclusion, the warlncRNAs signature for PAAD constructed with six lncRNAs not only has preferable prognostic value and prediction capacity but also contributes to risk stratification and predicts the immune efficacy and drug sensitivity of PAAD patients. In clinical practice, combining risk scores based on the expression levels of six lncRNAs with the age and stage N of PAAD patients may help them benefit from individualized therapy.

## Data Availability

All datasets used in this study can be obtained as indicated.

## Supplementary Materials

The following supporting information can be downloaded at: https://www.mdpi.com/article/10.3390/biology12020319/s1, Table S1: 354 autophagy- and Wnt/β-catenin-related lncRNAs; Table S2: Autophagy- and Wnt/β-catenin-related lncRNAs.
